# Role of Nutritional Habits during Pregnancy in the Developing of Gestational Diabetes: A Single-Center Observational Clinical Study

**DOI:** 10.3390/medicina60020317

**Published:** 2024-02-13

**Authors:** Jelena Trifunovic-Kubat, Predrag Sazdanovic, Milos Ilic, Djordje Filipovic, Tamara Nikolic Turnic, Sladjana Mihajlovic

**Affiliations:** 1 University Clinical Center “Dr Dragisa Misovic Dedinje”, Heroja Milana Tepica 1, 11000 Belgrade, Serbia; jelena.j.trifunovic@gmail.com (J.T.-K.); mmilosilic@gmail.com (M.I.); djordje.skijas@gmail.com (D.F.); drsladjanamihajlovic@gmail.com (S.M.); 2 Department of Gynaecology and Obstetrics, Faculty of Medical Sciences, University of Kragujevac, Svetozara Markovica 69, 34000 Kragujevac, Serbia; predrag.sazdanovic@gmail.com; 3 University Clinical Center Kragujevac, Clinic of Gynaecology and Obstetrics, Zmaj Jovina 30, 34000 Kragujevac, Serbia; 4 Department of Pharmacy, Faculty of Medical Sciences, University of Kragujevac, Svetozara Markovica 69, 34000 Kragujevac, Serbia; 5 NA Semashko Public Health and Healthcare Department, F.F. Erismann Institute of Public Health, I.M. Sechenov First Moscow State Medical University (Sechenov University), 119048 Moscow, Russia; 6 Faculty of Medicine, University of Belgrade, Dr Subotića 8, 11000 Belgrade, Serbia

**Keywords:** nutritional habits, gestational diabetes mellitus, pregnancy, validation of questionnaire

## Abstract

(1) *Background and Objective*: Excessive gestational weight gain is associated with serious complications such as pre-eclampsia, fetal macrosomia and a more frequent need for cesarean section. The aim of this study is to develop a simple screening model that includes maternal age, BMI and nutritive habits in the second trimester in order to predict the risk of GDM in the population of pregnant women in the territory of the Republic of Serbia. (2) *Materials and Methods*: This single-center, prospective and case–control study was performed in the University Clinical Center “Dr. Dragisa Misovic Dedinje”, Belgrade, Serbia and included 54 women with singleton pregnancies during the second trimester from July 2023 to November 2023. We used basic demographic and socio-epidemiological data, as well as data of the present comorbidities and previous pregnancies/births. The Serbian version of the Nutritive Status Questionnaire (NSQ) was used to estimate the nutritive habits in GDM (*n* = 22) and non-GDM groups (*n* = 32). (3) *Results*: We observed less frequent vegetable and fruit consumption in the GDM group in comparison with the non-GDM group; meat and chicken intake was 2–3 times per week in both groups; meat products were consumed 2–3 times per week in the GDM group and 2–3 times per month in the non-GDM group; milk products were consumed once a day in 31.8% of GDM patients and twice per day in 24.1% of non-GDM patients. Sweets (cakes, ice creams, biscuits) were consumed very often (2–3 times per week) in the GDM group (36.4%), while in the non-GDM group this habit was less frequent (26.7%). Cronbach alpha and internal consistency for this instrument were very good (Cronbach alpha = 0.87). (4) *Conclusions*: We have found that a non-adequate intake of fruits/vegetables, dairy and whole grain, as well as an excessive intake of sugar/artificially sweetened beverages and dairy, was associated with a higher risk of gestational diabetes mellitus (OR = 0.04; 95% CI).

## 1. Introduction

There are two types of gestational diabetes in women: gestational diabetes (including type 1 and type 2 diabetes) and gestational diabetes mellitus (GDM). In the 1950s, the term “gestational diabetes” was coined to describe a temporary condition in women that negatively affects fetal development and disappears after delivery [1]. GDM is insulin resistance that begins or is first recognized during pregnancy [2]. This diagnosis does not apply to pregnant women who have already been diagnosed with diabetes [3,4]. According to the International Diabetes Federation (IDF), one in seven pregnant women have been diagnosed with GDM [5]. Worldwide, the prevalence of GDM is estimated at around 15% according to a systematic review [6]. The prevalence of GDM ranges from 2–6% in most racial/ethnic groups studied to 10–20% in high-risk populations, with an upward trend in most racial/ethnic groups [7]. In 2019, a meta-analysis using the same criteria reported that the highest pooled prevalence (11.4%) of GDM was found in South Asia (Bangladesh, India and Sri Lanka) compared to the rest of the world (3.6–6.0%) and that South Asian and Hispanic individuals were at the highest risk [6,7].

Several models for screening the development of diabetes in pregnancy have been described in the literature [8,9]. One of the models, which combines maternal factors such as history of GDM, family history of diabetes, ethnicity, parity, BMI, mean arterial pressure (MAP), uterine artery pulsatility index (UtA PI) and pregnancy-associated plasma protein A (PAPP-A) in the first trimester, has low precision [7]. Another model developed using a software algorithm based on data from first trimester health records to predict the risk of GDM in the 24th to 28th week of pregnancy was used. This algorithm also considered maternal factors such as age, parity, BMI, education, FPG and other hematologic and biochemical test results [8]. In addition, a model for obese pregnant women was developed that included data on age, previous GDM, family history of type 2 diabetes, systolic blood pressure, sum of skinfold thickness, waist-to-height ratio and neck-to-thigh ratio, with an accuracy of approximately 72%. Currently, in the Republic of Serbia, an oral glucose tolerance test (OGTT) is recommended as a screening test for pregnant women who have not been diagnosed with type 1 or type 2 diabetes and who do not have symptoms of gestational diabetes. There is no international consensus on the timing of the screening method and the optimal cut-off points and questionnaires for the diagnosis and intervention of GDM [9,10,11].

Weight gain during pregnancy refers to weight gain from the time of conception to delivery. In 2009, the Institute of Medicine published guidelines for appropriate weight gain in pregnant women depending on previous body mass index, the number of fetuses and the current trimester of pregnancy [9,10,11]. Excessive weight gain during pregnancy is associated with serious complications such as pre-eclampsia, fetal macrosomia and a more frequent need for cesarean section. In addition, excess weight is more often maintained, which brings with it the risk of cardiovascular disease (hypertension, dyslipidemia, ischemic disease, stroke, etc.).

Various studies have shown that pregnant women who consume more fruits and vegetables, whole grains, fish and healthy fats and avoid foods with low nutritional value have a lower risk of excessive weight gain during pregnancy and associated pregnancy complications [5,12,13]. In addition, a growing number of studies suggest that hypertriglyceridemia is associated with GDM [6,7,8,9,10,11,12,13]. Hypertriglyceridemia itself is a known risk factor for metabolic syndrome and is independently associated with pregnancy outcomes such as low birth weight, macrosomia [6,7,8,9,10,11,12,13,14,15] and preterm birth [10]. It is not known whether and to what extent TG predicts GDM.

Various questionnaires have been proposed in the literature to assess the dietary habits, lifestyle or quality of life of the general population, but although the general questionnaires are valid, they may not be appropriate to measure the dietary habits or disorders of a specific population such as pregnant women or women with GDM [6,7,8,9,10,11,12,13,14,15].

Notwithstanding the fact that a healthy diet is very important for both the mother and the fetus, there is no suitable questionnaire in clinical practice that could quickly and effectively survey pregnant women about their dietary habits and then identify those at risk of excessive weight gain during pregnancy. It is hoped that this research will contribute to the development of a questionnaire that can identify pregnant women at risk of early diabetic complications and that healthcare professionals will devote more time and attention to educating pregnant women about appropriate nutrition.

The aim of this study is to develop a simple screening model that includes maternal age, BMI and nutritive habits in the second trimester in order to predict the risk of GDM in the population of pregnant women in the territory of the Republic of Serbia. Also, this study aims to construct a model that would help in clinical practice to recognize bad nutritional habits which could induce gestational diabetes or its complications.

## 2. Materials and Methods

### 2.1. Ethical Approval

All procedures involving human participants were performed by the ethical standards of the Institutional Review Board of the Dr. Dragisa Misovic tertiary hospital, Belgrade, Serbia (date 19 May 2023; number 4473/13-2023) and the 1964 Declaration of Helsinki and its later amendments or comparable ethical standards. Informed consent was obtained from all individual participants included in the study and no incentive was offered for participation in the study.

### 2.2. Study Design, Setting and Population

This single-center, prospective, observational clinical study was performed in the Dr. Dragisa Misovic tertiary hospital, Belgrade, Serbia. From July 2023 to November 2023, 54 women with singleton pregnancies and at at least 13 gestational weeks were included after a regular visit to the Department of Obstetrics. Inclusion criteria: adult pregnant women aged 18 years or older with a diagnosis of gestational diabetes mellitus (GDM group, *n* = 22) or none of the potential risk factors of GDM (non-GDM group, *n* = 32). Exclusion criteria were pre-existing diabetes mellitus, multiple pregnancy, use of medication that influences glucose metabolism (e.g., steroids, β-adrenergic agonists and antipsychotic drugs), physical disability or severe psychiatric disorders (schizophrenia, major depression, bipolar disorders, etc.).

### 2.3. Gestational Diabetes Mellitus (GDM) Criteria

GDM was defined according to the WHO 2013 and ADA guidelines and as one or more pathologic glucose values in a 75 g 2 h oral glucose tolerance test (OGTT) during pregnancy [16,17,18]. The diagnostic thresholds were as follows: fasting plasma glucose (FPG) ≥ 5.1 mmol/L, 1 h value ≥ 10.0 mmol/L and 2 h value ≥ 8.5 mmol/L. According to the values of the OGTT at 24 to 28 weeks of gestation, diagnosis of GDM was made. According to these criteria, all participants were divided into two groups: a group with gestational diabetes mellitus (GDM group) and a group without gestational diabetes mellitus (non-GDM group).

### 2.4. Data Collection and Estimating the Outcomes

All data were collected during the clinical examination and the visit to the Department of Obstetrics. Primary endpoints were the estimation of basic demographic and socio-epidemiological data, as well as data on the present comorbidities and previous pregnancies/births. The data that were collected included: age, race, cigarette and alcohol consumption before and during pregnancy, method of conception (spontaneous or assisted), medical history (presence of hypertension, diabetes, antiphospholipid syndrome, thrombophilia), medical history before and during pregnancy (use of antihypertensives, antidepressants, antiepileptics, aspirin, corticosteroids, insulin and thyroxine), parity (nulliparity with or without previous pregnancies or miscarriage before 24 weeks), previous pregnancy with diabetes, positive family history for diabetes. Additional data related to pregnancy that were collected are gestational week and time interval since the previous pregnancy (in months).

### 2.5. Nutritional Habits Assessment

All questionnaires were filled in during the visit of patients to the Department of Obstetrics in the doctor’s office as a self-assessment method. In this study, we used a standardized questionnaire [19] about the nutritive status which consisted of 15 questions divided into 6 domains: various foods, high-fat foods, beverages, bread and supplements. All questions are specific to examining the eating habits of pregnant women, such as the intake per day, week and month in portions in the last four weeks. According to the values of the questionnaire, we calculated dietary risk factors (predictors): not eating a varied diet, fruits/vegetables < 5 times per day, dairy < 2 times per day, whole grain products < 2 times per day, sugar/artificially sweetened beverages ≥ 5 times per week and dairy ≥ 5 times per day. The calculation for recommendation of the adequate diet was carried out in accordance with the Nordic Nutrition Recommendations [19]. 

### 2.6. Validation of Serbian Version of Nutritive Status Questionnaire (NSQ)

The NSQ was translated from English to Serbian following the WHO’s guide for translation and adaptation of instruments to be sure of linguistic and cultural equivalence between English and Serbian versions. The translation process was performed by forward and backward translation and review of cognitive interviews. Cognitive interviewing is a technique used to provide insight into participants’ perceptions in which individuals are invited to verbalize thoughts and feelings as they examine information. After the cognitive interviewing, there were no additional interviews since the questions required no modifications. The final step was proofreading and finalizing the Serbian version of the NSQ [19,20,21].

For the validation of the Serbian version of the NSQ, participants were randomly recruited from a study sample during the clinical study. All participants met the inclusion criteria defined prior to the study. Test–retest reliability between the first and second responses to the questionnaire was calculated for all items based on the infraclass correlation coefficient (ICC). An ICC below 0.50 reflected poor reliability, between 0.50 and 0.75 reflected moderate reliability and above 0.75 reflected good reliability. After that, we estimated internal consistency of the instrument where we measured reliability of items and separate domains. The Cronbach alpha coefficient with a range from 0 to 1 was calculated. Cronbach α values ranged from excellent (≥0.9), good (0.8–0.9), acceptable (0.7–0.8), questionable (0.6–0.7) and poor (0.5–0.6) to unacceptable (≤0.5) [21].

### 2.7. Statistical Analysis

Continuous variables with a normal distribution are presented as mean ± standard deviation and were compared using the Student *t*-test. All categorical variables are summarized and expressed as proportions and compared using the chi-square test with normal approximation or Fisher’s exact test, as appropriate. A logistic regression model was used to evaluate the effect of nutritive habits on the risk of GDM and adverse maternal outcomes. A significance level of 0.05 was set for retaining variables in the logistic regression model. All statistical analyses were performed using IBM SPSS version 26.0 for Mac (IBM Corp., Armonk, NY, USA).

### 2.8. Power Analysis

The sample size of this observational study is based on required power (80%) of the smallest sample size needed to obtain a significant result and it was carried out a priori. For an observational study, the maximum sample size is 10% of the population. Since at the University Clinical Center Kragujevac the total number of pregnant women was around 200–220, the minimum number of pregnant women included in the study must be 20–22 per group [22].

## 3. Results

### 3.1. Test–Retest Reliability and Validation of NSQ Instrument

Cronbach alpha and internal consistency for this instrument were very good (Cronbach alpha = 0.87). Also, the item internal consistency (IIC) for each item was high and above 0.75, which indicates that all items met the criteria and standards for internal consistency and represent an adequate tool. Also, exploratory factor analysis performed using the maximum-likelihood method (Supplement Table S1). We observed that factor analysis resulted in five factors with values around 1. According to the item structure (above 0.36) suggested by analysis, the factors fit into six subscales.

### 3.2. Anthropometric and Demographic Characteristics of Study Population

Basic data of the GDM and non-GDM groups are presented in Table 1. The mean age in GDM and non-GDM groups were similar, as well as gestational week and the number of spontaneous pregnancies. Regarding the anthropometric characteristics, the mean weight and height were similar, but the BMI was statistically significantly higher in the GDM group (Table 1). Most of the women in both groups were pregnant for the first time. 

Anamnestic data of both groups are presented in Table 2. Statistically significant differences in frequency of proteinuria, positive history for DM, hypertension, diabetes mellitus, thrombophilia and cigarette consumption were observed. Also, antihypertensive treatment and thyroxine administration were more frequent in the GDM group. Vaginal delivery was present in 73.7% in the GDM group, while in the non-GDM group this value was in 91.7% (Table 2).

### 3.3. Nutritional Habits in GDM and Non-GDM Groups

The frequency of intake of the main food groups by participants based on recommendations on gestational weight can be seen in Table 3. In the GDM group, fruits and vegetables were less frequently consumed in comparison with the non-GDM group; meat and chicken intake was 2–3 times per week in both groups; meat products were consumed 2–3 times per week in the GDM group and 2–3 times per month in the non-GDM group; milk products were consumed once a day in 31.8% of GDM patients and twice per day in 24.1% of non-GDM patients. Sweets were consumed very often (2–3 times per week) in the GDM group (36.4%), while in the non-GDM group this habit was less frequent (26.7%). Lipids such as oils, butter and similar products were more often consumed by GDM patients in comparison with controls: 22.7% consumed oils 2–3 times per week compared to 13.3% in the non-GDM group. High-fat milk was predominately consumed by GDM patients 2–3 times per week (22.7%) while it was consumed by 13.3% of patients 2–3 times per week in the non-GDM group. Very similar consumption of carbonated drinks with sugar was observed in both groups (1 per month in 45.5 and 43.3%). Also, coffee was consumed in 22.7% of GDM patients once per month and 27.6% of non-GDM patients. Regarding supplements, most participants in both groups took folic acid every day and vitamin D less than once a month and most of the pregnant women with or without GDM used a multivitamin supplement. It was found that, during pregnancy, 20.4% of pregnant women had never consumed fish, 13.1% abstained from red meat and 12.4% excluded white meat from their diet (Table 3).

The distribution of responses regarding the types of nutrients which are not recommended during pregnancy among the study population is presented in Table 4. Regarding the main groups of foods in the analysis and restriction, we have observed in the GDM group that fewer participants avoided cereals and food with a high fat content, but a large number of them avoided the consumption of fruits, fish, meat and grain products with high fat content in general (Table 4). 

Table 5 shows the logistic regression model for estimating the association between domains of the questionnaire (types of food as risk factors) and the presence of GDM and high body mass index. We have observed that high-fat food and bread had a positive association with body mass index, and with additional beverages these products had a positive correlation with onset of GDM. Also, a non-adequate intake of fruits/vegetables, dairy and whole grain, as well as an excessive intake of sugar/artificially sweetened beverages and dairy, was associated with a higher risk of gestational diabetes mellitus (OR = 0.04; 95% CI). Supplements do not have any significant influence on body mass index nor on metabolic disorders (Table 5).

## 4. Discussion

The aim of this study is to develop a simple screening model that includes maternal age, BMI and nutritive habits in the second trimester in order to predict the risk of GDM in the population of pregnant women in the territory of the Republic of Serbia. Also, this study has the purpose of constructing a model that would help in clinical practice to recognize bad nutritional habits that could induce gestational diabetes or its complications. We found that non-adequate nutritional habits regarding the type of food and excessive intake could be associated with a higher risk for metabolic disorders during pregnancy. Our results suggest that by asking simple questions about nutritional habits, we might be able to identify women with potential extreme weight gain and diagnose diabetes.

Literature data from many studies confirmed the significance of nutrition during pregnancy in developing gestational diabetes mellitus and its complications [23,24,25]. As we know, gestational diabetes (GDM) is defined as any degree of glucose intolerance with onset during pregnancy and is associated with increased feto-maternal morbidity as well as long-term complications in mothers and offspring [23,24,25]. Women detected to have diabetes early in pregnancy receive the diagnosis of overt, non-gestational diabetes (glucose: fasting ≥ 126 mg/dL, spontaneous ≥ 200 mg/dL or HbA1c ≥ 6.5% before 20 weeks of gestation). GDM is diagnosed by an oral glucose tolerance test (OGTT) or increased fasting glucose (≥92 mg/dL). Screening for undiagnosed type 2 diabetes at the first prenatal visit is recommended in women at increased risk (history of GDM/prediabetes; malformation, stillbirth, successive abortions or birth weight > 4500 g previously; obesity, metabolic syndrome, age > 35 years, vascular disease; clinical symptoms of diabetes (e.g., glucosuria) or ethnic origin with increased risk for GDM/T2DM (Arab, South and Southeast Asian, Latin American)) using standard diagnostic criteria [23,24,25]. Performance of the OGTT (120 min; 75 g glucose) may already be indicated in the first trimester in high-risk women but is mandatory from gestational weeks 24–28 in all pregnant women with previous non-pathological glucose metabolism.

On the other hand, to maintain a healthy pregnancy, approximately 300 extra calories are needed each day [26,27,28]. These calories should come from a balanced diet of protein, fruits, vegetables and whole grains. Sweets and fats should be kept to a minimum. Proper diet during pregnancy is one of the conditions for correct fetal development and the maintenance of good health in pregnant women [28]. Increased demand for energy and nutrient substances is connected with the growth of the mother’s tissue mass and also with the growth of fetal mass and afterbirth. During pregnancy, the quality and composition of the diet are not only important but also the quantity and regularity of meals taken [29]. 

In our study, we observed that vegetables and fruits were consumed less frequently in the GDM group in comparison with the non-GDM group; meat and chicken intake was 2–3 times per week in both groups; meat products were consumed 2–3 times per week in the GDM group and 2–3 times per month in the non-GDM group; milk products were consumed once a day in 31.8% of GDM patients and twice per day in 24.1% of non-GDM patients. Sweets were consumed very often (2–3 times per week) in the GDM group (36.4%), while in the non-GDM group this habit was less frequent (26.7%). Lipids such as oils, butter and similar products were more often consumed by GDM patients in comparison with controls: 22.7% consumed oils 2–3 times per week compared to 13.3% in the non-GDM group. High-fat milk was predominately consumed by GDM patients 2–3 times per week (22.7%), while it was consumed by 13.3% of non-GDM patients 2–3 times per week. Very similar consumption of carbonated drinks with sugar was observed in both groups (1 per month in 45.5 and 43.3%). Also, coffee was consumed by 22.7% of GDM patients once per month and in the non-GDM group by 27.6%. Regarding supplements, the majority of participants in both groups took folic acid every day and vitamin D less than once a month, and most pregnant women with or without GDM used multivitamin supplements (Table 3).

Furthermore, we have observed that, in the GDM group, fewer participants avoided cereals and foods with high fat content, but a large number of them avoided the consumption of fruits, fish, meat and grain products with high fat content in general (Table 4).

Table 5 shows results that confirmed the association between intake of some types of food, the presence of GDM and a high body mass index. We have observed that high-fat foods and bread have a positive association with body mass index, and with additional beverages, these products have a positive correlation with the onset of GDM. Supplements do not have any significant influence on body mass index or metabolic disorders (Table 5).

Our results confirmed the previous results of clinical trials that examined the role of food intake in weight gain and diabetes complications. Alfred and coworkers examined the nutritional status of pregnant women hospitalized in a department of obstetrics and gynecology. They followed 96 women who filled out the questionnaire about their diet during pregnancy. Results showed that nutrition habits do not change significantly during pregnancy and that pregnant women are not sufficiently informed about a healthy diet during pregnancy by doctors, which leads to irregularities in taking meals [30].

Definitely, pregnancy is a special period of increased nutritional needs during which conscious nutritional support is required. Insufficient and imbalanced nutrition in this period of life causes serious conditions that affect both child and mother. The screening methods for diabetes mellitus are very useful and effective, but the main question is how to control glycemia in an adequate manner during pregnancy and how to be sure in prognosis of weight gain. Currently, the traditional model is used for diagnosing GDM. Screening tests may vary slightly depending on the health care provider but generally include: initial glucose challenge test, where a blood sugar level of 190 milligrams per deciliter (mg/dL) or 10.6 millimoles per liter (mmol/L) indicates gestational diabetes [31,32]. A blood sugar level below 140 mg/dL (7.8 mmol/L) is usually considered within the standard range on a glucose challenge test, although this may vary by clinic or lab. Another test is follow-up glucose tolerance testing, which is similar to the initial test, except the sweet solution has even more sugar and blood sugar is checked every hour for three hours. In this test, if at least two of the blood sugar readings are higher than expected, gestational diabetes is diagnosed. Besides these tests, it is important to find a fast and non-invasive prediction test for GDM and one of them is the nutritional habit questionnaire used in this study. Since a healthy diet which focuses on fruits, vegetables, whole grains and lean protein—foods that are high in nutrition and fiber and low in fat and calories—and limits highly refined carbohydrates, including sweets, is recommended, this instrument must be focused on detecting poor nutritional habits in pregnant women. Research led by Tiffany et al. [33] showed that, regardless of the prepregnancy body mass index, controlling weight gain during pregnancy is of great significance for reducing the risk of maternal and fetal complications. 

In clinical practice, physicians can guide pregnant women to manage and control weight gain during pregnancy in order to reduce the risk of adverse pregnancy outcomes, and this instrument could be one of the guides for that purpose [34,35,36]. The Finnish Gestational Diabetes Prevention Study (RADIEL) suggests that the risk of GDM can be reduced by approximately 40% by following a combination of moderate physical activity and diet intervention among high-risk women [33]. Kondracki et al. examined the risk of large for gestational age (LGA) (≥97th percentile) singleton births at early term, full term and late term in relation to maternal prepregnancy BMI status mediated through GDM [36]. They concluded that health risks arise from prepregnancy overweight, which means that obesity can be a substantial risk to both mothers and their offspring [37].

Strengths of the present investigation were that data were collected from groups of cases/pregnant women and that outcome measures were prospectively assessed. This study provides a sensitive tool for estimating nutritive habits during pregnancy. Also, this questionnaire is a relatively cheap and quick way to gather a large amount of data. Highlights of this research lie in the validation of the first Serbian version of a nutritive habits assessment questionnaire, which could be good aid in ambulatory and hospital clinical practice. 

However, a few limitations should be taken into consideration. First, the sample size was not large, and there were no details about potential physical activity which could be a significant factor in reducing weight gain and a healthy lifestyle. Further cohort clinical studies are needed to include more variables and more pregnant women in order to estimate the all risk factors for gestational diabetes mellitus.

## 5. Conclusions

In conclusion, we found that inadequate intake of fruits/vegetables, dairy products and whole grains and excessive intake of sugar/artificially sweetened beverages are associated with a higher risk of gestational diabetes mellitus. Our findings are consistent with the fact that nutritional counseling to promote a healthy lifestyle and diet during pregnancy needs to focus not only on overweight and obese women but also on women of normal weight.

## Figures and Tables

**Table 1:** Basic demographic characteristics of study population. Chi-square test of differences among groups or Student *t*-test for continuous variables was carried out. Statistical significance level was set at 0.05.

Variables	GDM Group [*n* = 22]				Non-GDM Group [*n* = 32]				*p*
	Min	Max	Mean	[SD]	Min	Max	Mean	[SD]	
Age [years]	27	39	33.14	3.62	26	44	31.50	4.02	0.568
Weight [kg]	56	104	78.41	11.39	58	90	74.07	9.48	0.645
Height [cm]	160	179	168.14	5.04	157	181	171.00	5.68	0.771
Body mass index [kg/m^2^]	22	39	27.77	4.10	21	30	25.07	2.81	0.048
Time since previous pregnancy (weeks)	19	80	46.00	22.30	15	120	35.46	28.63	0.021
Gestational week	23	37	28.77	2.56	19	34	28.90	3.02	0.987
First pregnancy [%]	Yes 63.6		No 36.4		Yes 56.7		No 43.3		0.677
Spontanenous pregnancy [%]	Yes 95.5		No 4.5		Yes 93.1		No 6.9		0.721

**Table 2:** Anamnestic data of study population in relation to presence of GDM. Chi-square test of differences among groups or Student *t*-test for continuous variables was carried out. Statistical significance level was set at 0.05.

Variables	GDM Group [*n* = 22]		Non-GDM Group [*n* = 32]		*p*
	Yes (%)	No (%)	Yes (%)	No (%)	
Proteinuria	9.1	90.9	3.3	96.7	0.041
Previous pregnancy with DM	27.3	72.7	6.7	93.3	0.001
Positive history for DM	50	50	10	90	0.001
Cigarette consumption before pregnancy	59.1	40.1	30	70	0.032
Cigarette consumption during pregnancy	18.2	81.8	6.7	93.3	0.045
Alcohol consumption before pregnancy	22.7	77.3	23.3	76.7	0.543
Alcohol consumption during pregnancy	0	100	0	100	0.766
Conception (spontaneous/artificial)	95.5	4.5	93.1	6.9	0.675
Hypertension	90.1	9.9	3.3	96.7	0.002
Diabetes mellitus	22.7	77.3	6.7	93.3	0.003
Antiphospholipid syndrome	0	100	0	100	0.788
Thrombophilia	27.3	72.7	16.7	83.3	0.022
Antihypertensive drugs before pregnancy	4.5	95.5	3.3	96.7	0.554
Antihypertensive drugs during pregnancy	13.6	86.4	3.3	96.7	0.043
Antidiabetic drugs before pregnancy	18.2	81.9	0	100	0.032
Antidiabetic drugs during pregnancy	54.5	45.5	13.3	86.7	0.021
Hypolipemic drugs before pregnancy	4.5	95.5	4.5	95.5	0.989
Hypolipemic drugs during pregnancy	4.5	95.5	4.5	95.5	0.989
Antiepileptic drugs before pregnancy	0	100	0	100	0.999
Antiepileptic drugs during pregnancy	0	100	0	100	0.999
Aspirin before pregnancy	9.1	91.9	3.3	96.7	0.879
Aspirin during pregnancy	36.4	63.6	23.3	76.7	0.788
Corticosteroid drugs before pregnancy	0	100	3.3	96.7	0.789
Corticosteroid drugs during pregnancy	4.5	95.5	3.3	96.7	0.899
Insulin therapy before pregnancy	0	100	0	100	0.999
Insulin therapy during pregnancy	0	100	0	100	0.999
Tiroxine therapy before pregnancy	4.5	95.5	16.7	83.3	0.041
Tiroxine therapy during pregnancy	4.5	95.5	26.7	73.3	0.039
Blood type	A 40 O 0 B 0 AB 60		A 26. O 47.8 B 17.4 AB 8.7		0.766
Rh factor (positive/negative)	70	30	73.9	26.1	0.870
Type of delivery (vaginal/surgical)	73.7	26.3	91.7	8.3	0.045
Complications	5.3	94.7	13.3	66.7	0.038

**Table 3:** Nutritional habits among study population in relation to presence of GDM. Chi-square test of differences among groups or Student *t*-test for continuous variables was carried out. Statistical significance level was set at 0.05.

Type of Food	GDM Group [*n* = 22]										Non-GDM Group [*n* = 32]									
	Per Month			Per Week			Per Day				Per Month			Per Week			Per Day			
	<1	1	2–3	1	2–3	4–6	1	2	3–4	>5	<1	1	2–3	1	2–3	4–6	1	2	3–4	>5
Vegetables	0	0	4.5	0	18.2	40.9	9.1	22.7	4.5	0	0	0	3.4	3.4	6.9	17.2	20.7	27.6	20.7	0
Fruit	0	0	0	4.5	18.2	45.5	22.7	4.5	4.5	0	0	0	3.4	0	6.9	13.8	27.6	27.6	10.3	10.3
Low-fat fish	45.5	13.6	13.6	27.3	0	0	0	0	0	0	31	31	6.9	20.7	6.9	0	0	3.4	0	0
High-fat fish	31.8	27.3	13.6	27.3	0	0	0	0	0	0	31	20.7	10.3	37.9	0	0	0	0	0	0
Red meat	4.5	9.1	13.6	13.6	36.4	18.2	4.5	0	0	0	3.4	9.1	13.6	13.6	36.4	18.2	4.5	0	0	0
Chicken meat	9.1	0	4.5	18.2	50	13.6	4.5	0	0	0	9.1	4.5	0	18.2	50	13.6	4.5	0	0	0
Meat products, meat pies, sausages	18.2	4.5	18.2	31.8	27.3	0	0	0	0	0	10.3	13.8	27.6	13.8	17.2	6.9	6.9	0	3.4	0
Milk products	4.5	0	4.5	0	27.3	18.2	31.8	9.1	0	4.5	3.4	0	6.9	3.4	17.2	20.7	13.8	24.1	6.9	3.4
Cheese	9.1	0	4.5	4.5	36.4	13.6	27.3	0	0	4.5	3.4		6.9	24.1	24.1	20.7	10.3	6.9	3.4	0
Whole grain products except bread	9.1	9.1	22.7	9.1	27.3	18.2	0	4.5	0	0	17.2	3.4	13.8	13.8	24.1	10.3	6.9	0	0	10.3
Legume dishes, stone fruit or seeds (not in bread)	4.5	9.1	18.2	18.2	31.8	13.6	4.5	0	0	0	6.7	0	23.3	13.3	36.7	3.3	3.3	10	3.3	0
French fries or snacks	22.7	4.5	31.8	27.3	9.1	4.5	0	0	0	0	13.3	6.7	26.7	13.3	16.7	0	6.7	16.7	0	0
Cakes and/or biscuits	22.7	18.2	0	9.1	36.4	4.5	9.1	0	0	0	6.7	10	16.7	23.3	16.7	6.7	16.7	3.3	0	0
Sweets and/or ice cream	4.5	9.1	13.6	9.1	36.4	18.2	9.1	0	0	0	3.3	6.7	6.7	23.3	26.7	6.7	23.3	3.3	0	0
Edible oils and dressings for cooking	18.2	0	9.1	13.6	22.7	18.2	9.1	9.1	0	0	10	3.3	26.7	20	13.3	6.7	10	10	0	0
Butter and similar products	27.3	4.5	9.1	27.3	18.2	4.5	9.1	0	0	0	20	3.3	16.7	16.7	26.7	13.3	3.3	0	0	0
Margarine and similar products	68.2	4.5	9.1	9.1	9.1	0	0	0	0	0	36.7	6.7	23.3	23.3	0	6.7	3.3	0	0	0
Pure fruit juice	18.2	4.5	13.6	13.6	31.8	13.6	4.5	0	0	0	16.7	6.7	10	16.7	40	3.3	6.7	0	0	0
High-fat milk	31.8	0	4.5	18.2	22.7	18.2	4.5	0	0	0	40	3.3	13.3	13.3	10	13.3	3.3	3.3	0	0
Low-fat milk	54.5	9.1	4.5	0	22.7	0	9.1	0	0	0	60	10	6.7	6.7	10	3.3	3.3	0	0	0
Low-fat milk with vitamin D	77.3	22.7	0	0	0	0	0	0	0	0	76.7	13.3	0	3.3	3.3	3.3	0	0	0	0
Yogurt, sour milk and thick yogurt	9.1	4.5	4.5	0	18.2	36.4	22.7	4.5	0	0	6.7	3.3	6.7	3.3	16.7	23.3	23.3	6.7	0	10
Vegetable milk	72.7	13.6	9.1	0	4.5	0	0	0	0	0	56.7	23.3	6.7	3.3	10	0	0	0	0	0
Carbonated and non-carbonated drinks with added sugar	45.5	22.7	4.5	9.1	18.2	0	0	0	0	0	43.3	10	16.7	13.3	13.3	0	3.3	0	0	0
Carbonated and non-carbonated drinks with sweeteners	59.1	13.6	4.5	18.2	4.5	0	0	0	0	0	66.7	6.7	13.3	10	3.3	0	0	0	0	0
Coffee	22.7	0	4.5	0	4.5	13.6	40.9	13.6	0	0	27.6	0	3.4	3.4	10.3	3.4	10.3	37.9	3.4	0
Alcohol	90.9	0	9.1	0	0	0	0	0	0	0	80	10	6.7	3.3	0	0	0	0	0	0
Whole grain bread	18.2	0	4.5	0	27.3	18.2	13.6	13.6	4.5	0	17.2	3.4	27.6	17.2	13.8	3.4	3.4	6.9	6.9	0
Classic white and semi-white bread	40.9	9.1	4.5	4.5	22.7	4.5	0	13.6	0	0	10.3	0	13.3	13.8	13.8	27.6	6.9	10.3	3.4	0
Rye bread with added sugar	63.6	0	4.5	9.1	18.2	4.5	0	0	0	0	69	10.3	10.3	3.4	6.9	0	0	0	0	0
Cod liver oil	95.5	0	0	3.4	0	0	3.4	0	0	0	93.1	0	0	3.4	0	0	3.4	0	0	0
Vitamin D	59.1	0	4.5	4.5	4.5	0	27.3	0	0	0	41.4	0	6.9	20.7	10.3	20.7	0	0	0	0
Folic acid/folate/folicin	31.8	0	0	0	0	9.1	54.5	4.5	0	0	20.7	0	3.4	3.4	6.9	3.4	48.3	3.4	0	10.3
Iron	59.1	0	4.5	4.5	4.5	0	27.3	0	0	0	41.4	0	3.4	3.4	10.3	3.4	24.1	3.4	0	10.3
Multivitamin with vitamin A	90.9	4.5	0	0	4.5	0	0	0	0	0	69	0	6.9	3.4	3.4	0	6.9	0	0	10.3
Multivitamin without vitamin A	77.3	0	4.5	0	0	0	18.2	0	0	0	65.5	3.4	0	3.4	3.4	3.4	10.3	0	0	10.3
Other supplements	45.5	0	4.5	0	4.5	4.5	31.8	9.1	0	0	24.1	0	3.4	3.4	0	13.8	37.9	6.9	10.3	0

**Table 4:** Avoided types of food among study population in both groups. Chi-square test of differences among groups or Student *t*-test for continuous variables was carried out. Statistical significance level was set at 0.05.

Type of Food	Non-GDM Group [*n* = 32]	GDM Group [*n* = 22]	*p*
Do You Avoid a Certain Group of Foods?	Yes 40.9 No 59.1	Yes 40.9 No 59.1	
What Foods Do You Avoid?	(%)	(%)	
Cereal products	21.2	16.5	0.023
Vegetables	11.3	12.8	0.450
Fruits	2.4	5.3	0.032
Fish	17.4	20.1	0.044
Meat	10.3	5.5	0.021
Eggs	8.4	10.3	0.567
Foods with a high fat content	40.1	21.4	0.002
Milk products with high fat content	10.7	9.7	0.452
Grain products with high fat content	4.5	7.2	0.043
Fish high in fat	4.5	3.2	0.521

**Table 5:** The association between domains of questionnaire (types of food as risk factors) and presence of GDM and high body mass index (≥25 kg/m^2^). Logistic regression model, adjusted for maternal age, parity, smoking during pregnancy, presence of hypertension. * indicates significant associations.

Variables	Body Mass Index			Gestational Diabetes Mellitus		
	Crude OR [95% CI]	Adjusted OR [95% CI]	*p* Value Adjusted OR [95% CI]	Crude OR [95% CI]	Adjusted OR [95% CI]	*p* Value Adjusted OR [95% CI]
Not eating a varied diet	1.06 [0.87, 1.28]	1.04 [0.86, 1.26]	0.067	1.43 [0.77, 2.66]	1.39 [0.73, 2.62]	0.063
Various foods	1.27 [1.04, 1.55]	1.276 [1.03, 1.54]	0.078	2.04 [1.09, 3.83]	2.20 [1.14, 4.25]	0.059
High-fat foods	1.23 [1.001, 1.54] *	1.22 [1.002, 1.50] *	0.023	2.03 [1.15, 3.02] *	2.01 [1.13, 3.00] *	0.021
Beverages	1.11 [1.004, 1.67]	1.10 [1.002, 1.65]	0.088	2.08 [1.34, 3.24] *	2.10 [1.36, 3.28] *	0.018
Bread	1.34 [1.124, 1.54] *	1.32 [1.121, 1.56] *	0.039	2.25 [1.34, 3.21] *	2.27 [1.37, 3.23] *	0.030
Supplements	1.45 [1.134, 1.56]	1.41 [1.130, 1.50]	0.084	2.45 [1.65, 3.56]	2.55 [1.75, 3.66]	0.075

## Data Availability

All data are available by request to the corresponding authors.

## Supplementary Materials

The following supporting information can be downloaded at: https://www.mdpi.com/article/10.3390/medicina60020317/s1, Table S1: Factor analysis among all tested questions in UseSuppQ. Extraction Method: Principal component Factoring, Rotation Method: oblimin rotation with Kaiser Normalization. Asteriks (*) indicate that factor has opposite value.
