# Supplementary material for: Role of Nutritional Habits during Pregnancy in the Developing of Gestational Diabetes: A Single-Center Observational Clinical Study
Source: Medicina (Kaunas). 2024 Feb 13;60(2):317. doi: 10.3390/medicina60020317 (PMC10890587; doi:10.3390/medicina60020317)
Supplement: Supplementary file 1 [file medicina-60-00317-s001.zip › medicina-2827901-supplementary.pdf]

Supplementary Table S1. Factor analysis among all tested questions in UseSuppQ. Extraction Method: Principal component Factoring, Rotation Method: oblimin rotation with Kaiser Normalization. Asteriks (*) indicate that factor has opposite value.

Item	Factors			Chi square/p value
**Factor I (Non eating a varied diet)**	1	2	3	
Coffee	0.674	0.667	0.771	0.078
Alcohol	0.715	0.543	0.678	**0.003**
Vegetables	0.532	0.543	0.567	**0.005**
Fruit	0.322	0.463	0.665	**0.004**
Milk products	0.748	0.771	0.665	0.089
Bread	0.703	0.458	0.665	0.120
**Factor II (Various foods)**				
Low fat fish	0.311	0.456	0.667	0.078
Chicken meat	-0.456	-0.245	-0.431	**0.032**
Cheese	-0.376	-0.563	-0.341	**0.022**
Pure fruit juice	0.102	0.034	0.023	**0.011**
Low fat milk	0.234	0.235	0.043	**0.045**
Low-fat milk with vitamin D	0.345	0.143	0.109	0.051
Yogurt. sour milk and thick yogurt	0.102	0.133	0.108	**0.032**
Vegetable milk	0.311	0.145	0.156	**0.045**
**Factor III (High-fat foods)**				
High fat fish	0.544	0.433	0.345	0.067
Red meat	-0.359	-0.045	-0.032	0.069
Meat products. meat pies, sausages	0.712	0.567	0.432	0.764
French fries or snacks	0.189	0.113	0.117	**0.045**
Cakes and/or biscuits cakes	0.453	0.347	0.378	0.067
Sweets and/or ice cream	0.042	0.056	0.012	0.078
Edible oils and dressings for cooking	0.045	0.046	0.043	0.066
Butter and similar products	0.367	0.458	0.490	0.088
Margarine and similar products	0.001	0.005	0.007	**0.001**
High fat milk	0.005	0.009	0.007	0.057
Carbonated and non-carbonated drinks with added sugar	0.532	0.543	0.567	**0.005**
Carbonated and non-carbonated drinks with sweeteners	0.322	0.463	0.665	**0.004**
Cod liver oil	0.748	0.771	0.665	0.089
**Factor IV (Beverages)**				
Whole grain product. except bread	-0.373	-0.561	-0.347	0.082
Legume dishes. stone fruit or seeds (not in bread)	0.101	0.033	0.024	0.083
**Factor V (Bread)**				
Whole grain bread	0.042	0.056	0.012	0.078
Classic white and semi-white bread	0.045	0.046	0.043	0.066
Rye bread with added sugar	0.367	0.458	0.490	0.088
**Factor VI (Supplements)**				

Vitamin D	0.712	0.567	0.432	0.764
Folic acid/folate/folicin	0.189	0.113	0.117	**0.041**
Iron	0.453	0.347	0.378	0.067
Multivitamin with vitamin A	0.042	0.056	0.012	0.078
Multivitamin without vitamin A	-0.359	-0.045	-0.032	0.069
Other supplements	0.234	0.235	0.043	0.012
